# Follow-up in non-muscle invasive bladder cancer: facts and future

**DOI:** 10.1007/s00345-020-03569-2

**Published:** 2020-12-26

**Authors:** J. Alfred Witjes

**Affiliations:** Department of Urology, University Medical Center Nijmegen, Radboudumc (610), P. O. Box 9101, 6500 HB Nijmegen, The Netherlands

**Keywords:** Non-muscle invasive bladder cancer, Risk groups, Follow-up, Urinary markers, Costs

## Abstract

Patients with non-muscle invasive bladder cancer (NMIBC) have high recurrence and progression rates in spite of tumor resection and adjuvant instillation therapy. To detect recurrences and progression, these patients remain under frequent follow-up. Follow-up, however, is not well defined. Frequency and duration of follow recommendations are based on low levels of evidence, which is illustrated by clear differences in these recommendations per guideline, even when specified per risk group. Additionally, follow-up is recommended with cystoscopy and cytology in selected patients, which both have clear limitations. Fact is that follow-up in NMIBC is too frequent, with low levels of evidence and suboptimal tools, and it is patient unfriendly and costly. Improved cystoscopy techniques are unproven or impractical in the outpatient follow-up setting. Urinary markers have been around for decades, but never widely used in clinical practice. New (epi)genetic markers, however, could play a significant role in future follow-up of NMIBC. They have been shown to have very high negative predictive values for recurrences in follow-up of NMIBC, especially high-grade recurrences. Several studies suggested that these markers could be used to adapt follow-up cystoscopy frequency. What still needs study and confirmation is the cost-effectiveness of the use of these markers, which is highly dependent on health care costs per country and marker price. In all, however, implementation of these new urinary markers after confirmation of current results might significantly reduce patient burden and health care costs in the near future without reducing quality.

## Introduction

Even after contemporary treatment of non-muscle invasive bladder cancer (NMIBC), meaning an adequate (re-)resection and adjuvant instillation therapy, recurrence and progression rates remain high. A good estimate of these numbers was recently provided by Ritch et al. who found 5-year recurrence rates 57%, 67% and 77% and 5-year progression rates of 7%, 26% and 46% in low, intermediate and high-risk patients, respectively [1]. These numbers might be somewhat lower with for example maintenance BCG therapy: Cambrier et al. mentioned a highest 51.7% recurrence rate and 19.8% progression rate at 5 years in a mix of Ta-T1 bladder tumor treated with adjuvant 1–3 years of BCG [2]. Also enhanced endoscopy and a single postoperative instillation of chemotherapy in low and intermediate risk tumors [3], all strategies recommended in current guidelines [4, 5], have led to a significant reduction in predominantly recurrence rates, but still numbers remain high. Even with current standard treatment, the number of events, recurrence and progression, in these patients remains around 50% within 5 years. This obviously necessitates follow-up since recurrences are at least bothersome, and progression potentially life threatening.

## Facts in follow-up

In spite of the large number of patients experiencing NMIBC and the subsequent high number of follow-up visits (Ploeg et al. estimated that at any time point 2.7 million patients have a history of bladder cancer and are in follow-up [6]), the frequency and duration of follow-up remain unclear. Follow-up frequency is based on risk groups, and the advice of the EAU (European Association of Urology) and AUA (American Urological Association) are more or less similar: first follow-up 3 months after the initial treatment, and if no recurrence is seen follow-up is more or less yearly in low-risk patients, for high-risk patients it is 3 monthly follow-up for 2 years, extending into yearly follow-up after 5 years, and an in between schedule for intermediate risk patients [4, 5]. The level of evidence for these recommendations, however, is low. Apart from the low level of evidence for these recommendations, other factors than the risk may play a role in the decision about follow-up. Heijnsdijk et al., for example, concluded that with increasing age there are less quality adjusted life-years gained with follow-up for recurrent bladder cancer, suggesting a decreased follow-up intensity with increasing age [7]. That we are far from lowering follow-up intensity was illustrated by Han et al. [8]. They compared guideline recommendations to cystoscopic surveillance intensity in daily practice in low-risk NMIBC patients. In 75% (835/1135) of patients, an excess of 1846 cystoscopies was performed. They rightfully raised concern about this overuse, both with regard to quality and costs.

The duration is probably even more subject to variation and subjectivity. In low-risk patients, for example, the EAU recommends to stop follow-up after 5 years, the AUA suggests that that should be a shared decision between patient and physician, and the NICE recommends to discharge patients back to primary care when the patients remains free of recurrence at 3 and 12 months [9]. Which guideline is right? Two studies specifically targeted the long-term outcome of low-risk patients and found, apart from many recurrences, a 2% and 2.4% cancer-specific mortality in primary pT1 grade 1 tumors with a median follow-up of 67 months [10], and within 24 and 105 months follow-up [11], respectively. Two other studies looked at NMIBC patients in all stages after being free of recurrences after a period of five years. Both studies found considerable rates of recurrences after these five recurrence-free years of 10.8% ([12] and 14.9% [13]. It seems clear that the duration of follow-up, even in low-risk patients, remains a difficult issue.

What about the value of the tools we use for follow-up? Guideline follow-up recommendations imply use of cystoscopies (strong recommendation in the EAU guideline [4]) in all risk groups, and cytology and upper urinary tract imaging in selected patients (the EAU guideline specifically mentions the high-risk group [4]). However, also these tools for follow-up have clear limitations. We know that white light cystoscopy does miss lesions, as has been demonstrated in numerous (randomized) studies on enhanced cystoscopy, as well as studies in “real life” practice [14]. Also urinary cytology has limitations. Specificity for high-grade tumors is good, but cytology is useless in case of infections or bladder instillation therapy, and sensitivity is not good, certainly not in lower grade tumors. The EAU guideline, therefore, concludes that cytology cannot be used to “reduce the number of cystoscopy procedures” [4].

In all, the recommendations for frequency and duration of follow-up of NMIBC patients with cystoscopy and cytology has a low level of evidence if any, the intensity is too high, the quality of tools used has clear limitations, and subsequently it is unnecessarily invasive, patient unfriendly and costly. And that in a time frame where, due to shortages in BCG and MMC patients are likely to be undertreated or treated with lower doses or reduced schedules, with at least an anticipated increase in recurrence rates [15].

## Enhanced cystoscopy and imaging for follow-up

As mentioned above, white light cystoscopy has limitations with sensitivity for papillary tumors of 70–80%, which is lower in case of CIS (carcinoma in situ). These numbers are in trials, and for the studies performed with blue light in the setting of a transurethral resection, so in the operating room. Can we also use these techniques to improve our performance during outpatient follow-up, which usually is done with flexible instruments.

NBI (narrow band imaging) is easy to perform in an outpatient setting, since it does not require additional preparation and the specific equipment can be used both in the clinical as well as in the outpatient setting. The real advantage of NBI, however, remains to be proven, since published data are limited and not in agreement. Mukherjee et al. showed in a randomized controlled trial NBI to be better in tumor detection and resection as compared to white light [16], whereas an earlier and larger study did not show a difference in recurrence rate after 1 year of follow-up [17].

Fluorescence cystoscopy (FC), on the other hand, has shown improved detection, resection and recurrence rates, but is more challenging for an outpatient procedure since it requires an instillation with hexaminolevulinate (HAL) of approximately 1 h and a specific blue light flexible cystoscope, which is not widely available. Lotan et al. published a consensus paper stating that HAL FC could lead to improved clinical outcomes by earlier detection of recurrences in follow-up that are unresponsive to therapy [18]. This suggests a role for FC in follow-up, although depending on a formal cost–benefit analysis. The studies using flexible FC in follow-up have shown similar significant advantages in detection rates, between 20 and 35%, as studies on perioperative use of FC with rigid instruments [19]. The instillation and waiting time for HAL, however, pose logistical issues in an outpatient setting.

The consequence of better tools in follow-up, in this case better cystoscopy, has been shown to result in more confidence and less anxiety among patients and urologists, resulting in longer intervals between cystoscopies [20]. Another way to improve the sensitivity of cystoscopy might be the use of artificial intelligence (deep learning), which was shown to improve both sensitivity and specificity resulting in a better bladder cancer detection and resection [21].

Other ways of imaging bladder cancer have clear limitations. CT and MRI are time-consuming and costly. MRI has been suggested to be useful in staging (invasive) bladder cancer [22], but in follow-up of NMIBC, MRI and CT play no role. Ultrasound can also be used, but data are again limited. A small study in 25 patients in follow-up of NMIBC showed a sensitivity and specificity of ultrasound of 84.6% and 91.7%, respectively, with flexible cystoscopy as the gold standard [23]. Tan et al. studied ultrasound and CT as screening tool in over 200 patients with microscopic hematuria [24]. “Optimal” bladder ultrasound had a sensitivity, specificity, and NPV for bladder cancer of 63.6%. 99.3% and 97.9%, respectively. For “optimal” CT urography these figures were 83.6%, 97.0% and 97.9%, respectively. In spite of the promising results, the authors concluded that for diagnosis imaging was not able to replace cystoscopy.

## Urinary markers

Bladder cancer is an ideal tumor for follow-up through markers in urine. Many have been tested and reported, only a few have been used in clinical practice. Urovysion™ (Abbott laboratories) is a fluorescent in situ hybridization (FISH) test that detects aneuploidy of chromosome 3, 7 and 17 and loss of 9p21. A systematic review (14 studies with 2960 patients) showed a sensitivity for bladder cancer of 76% (95% confidence interval (CI) 65–84%), and a specificity of 85% (95% CI 78–92%) [25]. This test is technically challenging and expensive, but it has been suggested that this test can predict response to BCG treatment [26], future tumour recurrences in high-risk NMIBC [27], and even progression of bladder cancer [28]. A simple and quick and cheap point of care test that has been used is the Nuclear Matrix Protein (NMP) 22 test (Matritech). Nucleair matrix protein is released after cell death, and tumour cells have an 80 times higher NMP22 concentration than benign cells. Therefore, an elevated NMP22 is associated the presence of bladder cancer. The review mentioned above found for NMP22 (41 studies, 13.490 patients) a sensitivity of 68% (95% CI 62–74%), and a specificity of 79% (95% CI 74–84%). For both tests, this means a better sensitivity as compared to cytology, but a lower specificity, and consequently these markers, as have been others, have not been considered sufficient to replace cystoscopy [4, 29]. Other issues with urinary markers are the impact of benign conditions such as urinary tract infections and the use during intravesical therapy.

Recently several interesting new urinary markers have been tested, based on genetic abnormalities or epigenetic changes which are frequent in bladder cancer, such as aberrant DNA methylation and non-coding RNA’s. Several of these tests have been studied in follow-up of NMIBC patients with very high negative predictive values (NPV’s) for (high grade) NMIBC recurrences and the potential to adapt follow-up policy.

In 2017, test results in over 1000 patients were published using a combination of five urinary gene expression markers and clinical data to rule out recurrences in patients during follow-up of NMIBC [30]. The combination in this prospective study had a sensitivity of 93%, (95% for high grade or T1 disease) and a NPV of 97%. Similar findings were reported in almost 1,000 patients from an international multicenter prospective study using a combination of urinary FGFR3, TERT and OTX1 during follow-up of NMIBC patients [31].

In 2018, a prospective study on a urinary test using hypermethylation was published [32]. A combination of 15 complementary proprietary DNA methylation biomarkers (Bladder Epicheck, Nucleix, Rehovot, Israel) was tested in NMIBC patients during follow-up. This was a one visit prospective multicenter study, and the Bladder Epicheck test was compared to cytology and cystoscopy results, with pathology as final confirmation of recurrent tumor. Overall sensitivity, specificity and NPV were 68.2%, 88.0% and 95.1%, respectively. However, excluding low-grade Ta recurrences, the sensitivity and NPV were 91.7% and 99.3%, respectively. Test results were independent of infections or intravesical therapy. These results were confirmed by an extension cohort, bringing the total number of patients tested above 600, with a NPV of 98.8% for non-high-grade recurrences [33], and e recent review of published evidence [34].

Another prospective study was published in 2019 where results of the Xpert (Cepheid, Sunnyvale, CA, USA) bladder cancer monitor test were described [35]. This test measures five mRNA targets (ABL1, CRH, IGF2, UPK1B, and ANXA10) that are frequently overexpressed in bladder cancer with a 90 min turnaround time. Also this study was performed in patients in follow-up after an episode of NMIBC, and test results were compared to cytology and the FISH test. Overall sensitivity of the Xpert test was 74%, 83% for high-grade tumors, and specificity was 80%. The NPV was 93% overall and 97.6% for HG tumors, which outperformed both cytology and the FISH test.

A retrospective analysis of pooled data on the performance of the Cxbladder test was also published in 2019 [36]. Cxbladder (Pacific Edge Ltd, Dunedin, New Zealand) is a quantitative mRNA test that measures five genes in unfractionated urine. In all, 852 samples from patients with haematuria or previously diagnosed urothelial carcinoma from 4 studies, which had Cxbladder results and cytology results, showed a NPV of Cxbladder of 97% (95% CI 94–98%) compared to 93% (95% CI 91–94%) for cytology. Additionally, Cxbladder correctly judged all patients diagnosed with urothelial carcinoma in case of atypical cytology and equivocal cystoscopy.

This year the diagnostic accuracy of a novel test targeting MCM5, a DNA licensing factor and biomarker of cell proliferation, was published, the ADXBLADDER test (Arquer Diagnostics, Sunderland) [37]. It was a prospective, multicentric European study for the detection of recurrence after NMIBC in the preceding 2 years. Recurrence was found in 127/1431 patients, and the ADXBLADDER test showed an overall sensitivity of 44.9% which was 75.6% for non-pTaLG tumours, and a specificity of 71.1%. The overall NPV was 93%, which was 99.0% for non-pTaLG recurrences. Even in the diagnostic setting in patients presenting with hematuria, the NPV of the ADXBLADDER test was 96.4%, 99.8% in non-pTa tumor [38].

A comparison of the four largest studies with prospective patient inclusion and markers that are currently marketed is given in Table 1

**Table 1:** Data of four largest studies with prospective patient inclusion

Test	Patient number	Tumor positive	Prospective	Overall			Non-low grade	
				Sensitivity (+ CI)	Specificity (+ CI)	NPV (+ CI)	Sensitivity (+ CI)	NPV (+ CI)
Bladder Epicheck [32]	353	46 (13%)	Yes	68.2% (52.4–81.4)	88.0% (83.9–91.4)	95.1% (91.9–97.3)	91.7% (73.0–99.0)	99.3% (97.4–99.9)
Bladder Epicheck extension [33]	657	80 (12.2%)	Yes	62.5%	85.8%	94.3%	86.4%	98.8%
Xpert [35]	239	43 (18%)	Yes	74% (60–85)	80% (73–85)	93% (89–96)	83% (64–93)	98% (49–99)
ADXBLADDER [37]	1431	127 (8.9%)	Yes	44.9% (36.1–54)	71.1% (68.5–73.5)	93% (91.2–94.5)	75.6% (59.7–87,6)	99% (98.2–99.5)

*NPV *negative predictive value, *CI* is confidence interval

A final new test for detecting NMIBC recurrence was also published this year: the Uromonitor-V2® test (U-monitor, Porto, Portugal), a urine assay based on the detection of hotspot alterations in three different genes (TERT, FGFR3 and KRAS) for detecting disease recurrence [39]. In all, 97 subjects participated in this prospective, single-visit, case-enriched cohort study. Twenty patients were controls, 29/49 had a history of NMIBC with/without current disease recurrence at time of enrollment. The test sensitivity, specificity and NPV were 93,1%, 85.4% and 95.3%, respectively. For cytology, this was 26.3%, 90.9% and 68.2%, respectively.

For all of the tests mentioned above, except the Cxbladder test, the authors explicitly suggested to adapt cystoscopy frequency based on the test result.

A comparison between two of the above-mentioned tests and cytology was published in 2020 [40]. In a cohort of 432 patients, 92 (21.3%) experienced NMIBC recurrence (54 low grade, 38 high grade). The overall sensitivity for low- and high-grade tumours was 13% and 47.4% for cytology, 53.7% and 79% for the Bladder EpiCheck test and 57.4% and 79% for the Xpert test, respectively. Overall specificity was 98.8% for cytology, 82.1% for Bladder EpiCheck and 76.5% for the Xpert test. The overall NPV’s were 83.6% for cytology, 89.4% for both the Bladder EpiCheck and the Xpert test. For both tests results quite similar to the above-mentioned studies, resulting in a similar statement about reduction of invasive follow-up in NMIBC patients.

Although some of these tests, like the ones in Table 1, are now available for clinical use, the future will learn whether their very high NPV will be acceptable for patients and sufficiently useful for urologists. Certainly, patient’s demands are high as recently again was described in a multicenter observational study in patients that had undergone cystoscopies and biomarker testing [41]. Patients value the seemingly high sensitivity of cystoscopy despite its discomfort, and before accepting any urinary test during surveillance instead of cystoscopy, test sensitivity should be similar to cystoscopy. As mentioned above, apparently the sensitivity of white light cystoscopy is by far not 100%, meaning that proper patient education might increase acceptance.

## Other options during follow-up

Since 2019, the EAU guideline has a recommendation on outpatient fulguration or laser vaporization of small papillary recurrences in follow-up of patients known with previous low-grade Ta tumors [4]. Although the recommendation is weak, this is often common practice in daily routine. Outpatient fulguration obviously does not prevent a cystoscopy, but it does reduce subsequent therapeutic burden.

Another option that reduces therapeutic burden is active surveillance (AS). As common as it is in prostate cancer and small renal masses, as unknown it is in NMIBC. The great advantage of NMIBC in follow-up, however, is that previous stage and grade are known, recurrences usually have the same stage and grade, which makes that urologists are able to predict stage and grade of a recurrence on cystoscopy rather well. This was even shown in the primary setting, where NMIBC was predicted accurately in 93.4% of cases [42]. Knowing the rather “benign” course of low-risk NMIBC, patients could be safely followed without direct intervention, thereby reducing patient burden and costs. Hurle et al. demonstrated safety and cost saving in a prospective study where low-risk patients (a history of pTa or pT1a low-grade tumors) were put on AS in case of a small recurrence without hematuria or positive cytology [43]. Median time on AS was 11 months with clear costs savings, and in case of subsequent resection progression in grade and stage was minimal and no patient developed MIBC. In a cohort of 106 patients on AS, the same authors found that the Xpert bladder cancer monitor only missed 2/22 patients requiring treatment in follow-up, and both were low-grade tumor, concluding that this test seemed reliable enough to avoid cystoscopies in the AS setting without missing high-grade tumors [44].

## Cost issues and follow-up

Finally there is a cost issue with bladder cancer and follow-up. Leal et al. looked at the economic costs of bladder cancer across the European Union (EU) [45]. These were €4.9 billion in 2012, of which health care costs were €2.9 billion (59%), productivity loss €1.1 billion (23%) and informal care costs €0.9 billion (18%). Bladder cancer costs represented 5% of total health care cancer costs and 3% of all cancer costs in the EU in 2012. Of note, difference between the least (Bulgaria) and most expensive country (Luxembourg) was > tenfold. For follow-up costs, all three fields (health care costs, productivity loss and informal care costs) play a role. Interestingly, Mossanen et al. used a Markow model to specifically evaluate costs of surveillance of NMIBC [46] Their index patient was a compliant 65-year-old male, and they used four health states: no evidence of disease, recurrence, progression/cystectomy and death. The cumulative costs over a 5-year period were USD 52,125, 146,250 and 366,143 for low-, intermediate- and high-risk, respectively. Costs for recurrence (= follow-up) were highest in the low-risk group, but still not more than 8% of the total costs of low risk. Progression and subsequent treatment results in much higher costs (71–92% depending on the risk group), even when not frequent in low-risk patients.

There have been prior cost-economic publications on the use of markers in surveillance of bladder cancer. Lotan et al. for example, used a decision analytical approach to evaluate alternating a tumour marker with cystoscopy and/or cytology [47]. They concluded that this approach is cost-effective for a wide range of marker sensitivities, specificities and costs, cystoscopy and/or cytology cost, a 20–80% yearly recurrence and a 2–40% yearly progression rate, provided marker cost are reasonable. This conclusion obviously is not very specific.

In all, there is certainly an opportunity to reduce patient burden and costs in an adapted follow-up strategy with a marker with a high NPV, where the financial benefit obviously depends on the price of the marker. Prospective studies will be necessary to validate these assumptions on national levels.

## Conclusion

Even if NMIBC patients are treated according to current guideline recommendations, recurrence and progression rates are high, with up to 50% of patients experiencing such an event in 5 years. This necessitates frequent follow-up. Follow-up, however, is not well defined. Recommendations on frequency and duration of follow-up of NMIBC patients differ per risk group, differ per guideline and have a low level of evidence. Tools for follow-up are cystoscopy and cytology, which have clear limitations. Actually, we do too much, of low quality and with low level of evidence, and it is patient unfriendly and costly. Enhanced cystoscopy improves tumor visualization, but still requires an invasive procedure in follow-up. New urinary markers could play a significant role in future follow-up of NMIBC, since several new markers have shown close to 100% NPV’s in follow-up for anything worse than low-grade NMIBC, and authors have suggested to adapt cystoscopy frequency based on the test result. Other developments might be active surveillance in recurrent low-risk NMIBC patients or outpatient fulguration, both reducing burden. Finally, it makes sense that the use of markers is cost-effective apart from being patient friendly, but this should be studied further before conclusions can be drawn.
